# Encapsulation of Curcumin With Persian Gum and Its Application to the Production of Functional Yogurt: Physicochemical, Antioxidant, Sensory Properties, and Starter Bacteria Survival Study

**DOI:** 10.1002/fsn3.4499

**Published:** 2024-10-07

**Authors:** Maziar Taghadosi, Marzieh Bolandi, Homa Baghaei

**Affiliations:** ^1^ Department of Food Science and Technology, Damghan Branch Islamic Azad University Damghan Iran

**Keywords:** antioxidant activity, curcumin, dairy product, encapsulation, stability

## Abstract

This study aimed to evaluate the ability of Persian gum to encapsulate curcumin and enhance its stability in dairy products. Given the increasing interest in functional foods, this study investigated the incorporation of nanocapsules containing curcumin (CLN) into functional stirred yogurt (CLN‐Y). CLN was prepared with Persian gum (PG) at different levels and included in the yogurt formula (CLN‐Y1%, CLN‐Y2%, and CLN‐Y3%). The physicochemical properties, rheological characteristics, antioxidant activity (AA), survival of starter bacteria, and sensory properties of the yogurt were evaluated over a 21‐day storage period at 4°C. The results showed that CLN significantly improved AA (reduction of 7.31% in the control vs. 1.85% in CLN‐Y3%), viscosity, hardness, and water holding capacity (reduction of 2.27% in control vs. 1.14% in CLN‐Y3%), while reducing syneresis (an increase of 10.87% in control vs. 3.16% in CLN‐Y3%) during storage (*p* < 0.05). CLN concentration directly affected AA. Although CLN‐Y exhibited a yellowish color and lower light intensity than the control, the samples were well accepted during storage. Increasing CLN levels led to decreased taste, color, and overall acceptance scores, and a 3% concentration is recommended for yogurt formulation due to its potential to improve yogurt quality, provide antioxidant benefits, maintain probiotic viability, and achieve high consumer acceptance during storage.

## Graphical abstract

Application of CLN (curcumin‐loaded nanoemulsion) to obtain the functional stirred yogurt was conducted successfully to increase antioxidant activity of final product.

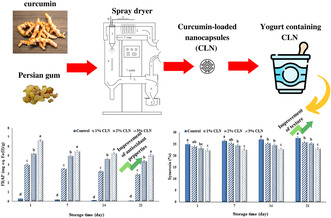

## 1 Introduction

Traditional dairy products like yogurt are classified as fermented foods and are made mostly by inoculating milk with lactic acid bacteria to initiate the fermentation process (Xiong et al. 2022). Common bacterial strains utilized in the production of yogurt include *Lactobacillus delbrueckii* subsp. *Bulgaricus* and *Streptococcus thermophiles* (Xiong et al. 2022; Zhao et al. 2023). Presently, there is a growing emphasis on functional products that possess inherent health‐promoting properties. Consequently, consumers are exhibiting a heightened demand for these products (Abedinia et al. 2021). Foods enriched with polyphenols and other natural bioactive compounds are among the most popular functional food products (Gao et al. 2022; Durmus, Capanoglu, and Kilic‐Akyilmaz 2021; Selahvarzi et al. 2021). Yogurt, apart from exhibiting diverse health advantages attributed to the presence of metabolites produced by *lactic acid bacteria* and the active constituents found in milk (such as bioactive peptides), can serve as an ideal substrate for the formulation of functional foods that yield amplified health impacts (Gruskiene, Bockuviene, and Sereikaite 2021).

Curcumin, acknowledged as a bioactive food compound, is employed in formulating functional food products. It is a prominent fat‐soluble polyphenolic compound derived from turmeric, displaying a vibrant yellow color and finding extensive use as a preservative, coloring agent, and food additive in diverse food products. Curcumin is attributed with a range of health benefits, encompassing antioxidative, antifungal, antibacterial, anti‐inflammatory, antidiabetic, and anti‐Alzheimer's activities (Meena et al. 2021). Despite the notable health advantages linked to curcumin, this active compound displays insufficient water solubility, low stability, and restricted bioavailability. Furthermore, it degrades easily in the presence of heat, UV light, and gastrointestinal conditions, which limits its use in food products (Chen et al. 2020; Guo et al. 2020). Encapsulation has been suggested as a prominent and effective approach to enhance the stability and bioavailability of bioactive compounds, such as curcumin (Azarashkan et al. 2022).

Encapsulation technology refers to a process wherein active agents are encapsulated within a wall material, allowing for controlled and sustained release. Hydrocolloids, proteins, carbohydrates, or lipids are commonly utilized as wall materials to coat bioactive compounds (Abedinia et al. 2020; Guo et al. 2024). Gums are polysaccharides that exhibit hydrophilic properties and are non‐toxic, easily accessible, biodegradable, odorless, tasteless, and colorless. Upon exposure to water, they form viscous solutions or gels capable of enveloping the active compounds (Taheri and Jafari 2019). PG, scientifically known as Zedo gum, is a translucent, odorless, and slightly turbid polysaccharide obtained naturally from wild almond trees indigenous to Iran. This gum exists in multiple forms, such as crystals, powder, and large granules, with colors ranging from white to reddish brown (Dabestani et al. 2018). PG is utilized either independently or in conjunction with other coating materials, such as chitosan and chickpea protein isolate, for the encapsulation of extracts and active compounds (Ershadi et al. 2021; Sarabi‐Aghdam et al. 2021).

The aim of this study was to investigate the effect of encapsulated curcumin on physicochemical, textural, antioxidant, and sensory properties of stirred yogurt as well as viability of starter bacteria of yogurt.

## 2 Materials and Methods

### 2.1 Preparation of CLN

A coating solution was prepared by combining 1.5 mg of PG with 20 mL of distilled water and incubating it at 60°C for 24 h. Curcumin powder, dissolved in pure ethanol at 1 mg/mL, was then added to the coating solution at a ratio of 1% v/w, along with 1% v/v of 80% tween. The mixture was thoroughly stirred until uniform and subsequently centrifuged at 10000 rpm for 15 min using a Froilabo centrifuge (France) (Zahrani et al. 2017). The capsule solution was dried using a spray dryer (Mini Spray Dryer B‐290, Germany) under the following conditions: inlet temperature of 120°C, nozzle diameter of 5–8 mm, feeding speed of 6.6 mL/min, dry air flow of 42–45 L/h, 90% aspiration, and pump speed of 20%.

#### 2.1.1 Investigation of the Characteristics and AA of CLN

The particle size, polydispersity index (PDI), and zeta potential of nanocapsules were determined using laser diffraction device (Zetasizer). To determine the particle size and PDI, 1 g of the powder sample was dissolved in 1 mL of 2‐propanol solvent and then a few drops of the obtained solution were injected to the device. To measure the zeta potential of nanocapsules, the samples were first prepared by diluting 10 μL of sample solution with 2 mL of deionized water. The encapsulation efficiency (EE) of nanocapsules was obtained by measuring the initial curcumin (C1) and the curcumin contained in the nanocapsules (C2) and through the following Equation (1) (Fan et al. 2018):

(1) $$\mathrm{EE}\;\left(\%\right)=\frac{C1-C2}{C1}\times 100$$

The morphology of curcumin nanocapsules was examined using a scanning electron microscope (SEM), and for this purpose, the nanocapsule first coated with gold using a scatter device for 5 min and then imaged at 15 mV magnification (Azarashkan et al. 2022). The AA of curcumin nanocapsules was investigated using two methods, including DPPH (2, 2 diphenyl‐1‐picrylhydrazyl) radical scavenging activity (Gorzin et al. 2024) and Fe III reducing power (Selahvarzi et al. 2021).

#### 2.1.2 Investigating the Retention Percentage of CLN

To find out if PG coating can retain both free and nano‐encapsulated curcumin, 4 mg/mL of free or encapsulated curcumin dissolved in ethanol was completely dispersed in a dialysis tube in phosphate buffer with pH = 7. After being placed in the dissolution machine, it was immersed in 200 mL of phosphate buffer (pH = 7) together with 80% w/v tween (0.5%) and stirred at 150 rpm at 37°C. The initial amount of curcumin in all samples was set at 100%. During the 4 h of the test, at certain times, a portion of the sample was removed and swapped out for an equal volume of phosphate buffer. Finally, the amount of curcumin in the medium was measured by RP‐HPLC system at a wavelength of 420 nm (Fan et al. 2018).

### 2.2 Fortification of Stirred Yogurt With CLN

To prepare the yogurt samples, the milk was initially heated to 90°C for 15 min and then cooled to the inoculation temperature of 45°C. The milk was then inoculated with yogurt starter bacteria (*Streptococcus salivarius* ssp. *thermophilus* and *Lactobacillus delbrueckii* ssp. *bulgaricus*) at a concentration of 3%. CLN was also added at three levels: 1%, 2%, and 3% w/w. The inoculated samples were poured into small containers and incubated at 42°C for 3 h until reaching a pH of 4.6. After the incubation period, the specimens were chilled to 4°C and preserved overnight. The yogurt clots were then broken by stirring at a low speed, and the samples were kept for 21 days in a refrigerator. Tests were performed on the yogurt samples every 7 days (Akgün et al. 2020).

### 2.3 Measurement of pH and Acidity

Using a pH meter (Mettler‐Toledo, Switzerland), the pH of the yogurt samples was measured at 20°C. The acidity of specimens was calculated using the titration method, which involved weighing a 10 mL sample, adding an equal weight of distilled water, adding 0.5 mL of phenolphthalein, and titrating with 0.1 N sodium hydroxide until a pink color appeared. The acidity was then calculated using Equation (2), where N is the amount of sodium hydroxide used and M is the sample weight (Senadeera et al. 2018).

(2) $$\text{Acidity}\;\left(\%\text{Lactic acid}\right)=N\times 0.009/M\times 100$$

### 2.4 Analyzing the Starter Bacteria's Vitality

To enumerate the yogurt starter bacteria, first, prepare the desired dilution of each sample using 0.1% peptone water. The M17 broth medium was used for *S. thermophiles*, and the MRS broth medium was used for *L. bulgaricus*. Plates for *Streptococcus* were incubated under aerobic conditions at 37°C for 48 h, while plates for *Lactobacillus* were incubated under anaerobic conditions at 37°C for 72 h. Finally, the number of formed colonies was counted and reported as log CFU/g (Senadeera et al. 2018).

### 2.5 Measurement of Syneresis and Water Holding Capacity (WHC)

Centrifugation (Froilabo, France) at 4°C and 16,125 × *g* for 20 min was used to remove 30 g of each yogurt sample in order to measure the syneresis of the samples, and the resulting whey was measured. Subsequently, the amount of syneresis was determined following Equation (3), where M1 represents the weight of the initial yogurt before centrifugation (g), and M2 denotes the mass of whey after centrifugation (g) (de Campo et al. 2019).

(3) $$\text{Syneresis}\;\left(\%\right)=M_{1}/M_{2}\times 100$$

To identify the samples' water holding capacity (WHC), 20 g of yogurt was transferred into a falcon tube and centrifuged for 10 min at 10732 × *g* at 4°C. After centrifugation, the whey was weighted, and using the following Equation (4), WHC of the samples was obtained, in which W1 and W2 were the initial weight of yogurt (g) and the weight of whey after centrifugation (*g*), respectively (de Campo et al. 2019).

(4) $$\mathrm{WHC}\;\left(\%\right)=M_{1}-M_{2}/M_{1}\times 100$$

### 2.6 Determining Apparent Viscosity

A Brookfield viscometer (USA) was used to evaluate the apparent viscosity of the samples at 4°C, and spindle number of 5 with shear rate of 60 rpm was used for this purpose. The viscosity number was recorded after 30 s (Sahan, Yasar, and Hayaloglu 2008).

### 2.7 Evaluation of Color Parameters

Color parameters of yogurt samples including lightness (L*), redness‐greenness (a*), and yellowness‐blueness (b*) were measured by Hunterlab colorimeter (Minolta, Japan). The yogurt samples of the same thickness (0.5 mm) were poured into a container of device and tested (Dinkçi et al. 2021).

### 2.8 Evaluation of Hardness

The texture analyzer (Stable Micro Systems, Surry, England) was used to test the samples' hardness at 5°C. A 100 g of sample was placed in the container without mixing, and a cylinder with a diameter of 20 mm, a penetration depth of 70 mm, cell bar of 0.1 N, and speed of 1 mm/s was used to compress the sample, and the hardness of texture was recorded (Karnopp et al. 2017).

### 2.9 Evaluation of AA

To calculate the AA of yogurt samples, 5 g of yogurt was combined with 20 mL of methanol twice, centrifuged at 4500 × *g* for 10 min, and filtered using filter paper. The resulting extracts were mixed twice, centrifuged for 15 min, and filtered to obtain the required extracts. Two methods were used to measure the AA of the samples: ferric‐reducing antioxidant power (FRAP) and 2,2‐diphenyl‐1‐picrylhydrazyl radical scavenging (DPPH). In order to measure the amount of DPPH radical scavenging, 0.5 mL of the sample's methanolic extract was combined with 4 mL of a 1 M DPPH methanolic solution. After adding distilled water, the mixture's volume was increased to 10 mL. After 30 min of room temperature storage in a dark, the mixture's absorbance at 517 nm was ultimately measured. Equation (5) was utilized to determine DPPH radical scavenging. The absorbance of the control and sample is represented by the letters Ac and As, respectively (Ahmad et al. 2021):

(5) $$\text{DPPH}\;\left(\%\right)=A_{c}-A_{s}/A_{c}\times 100$$

To determine AA by FRAP method, 1 mL of methanolic extract of sample was mixed with 2.2 mL of phosphate buffer and 2.5 mL of potassium ferrocyanide and placed in a water bath at 50°C for half an hour. Then 2 mL of 10% w/v trichloroacetic acid was added to it, and the resulting mixture was centrifuged at 3000 rpm for 10 min. The absorbance of the mixture was recorded at 700 nm (Selahvarzi et al. 2021).

### 2.10 Sensory Evaluation

Ten trained panelists, aged 25–45, assessed the sensory qualities of yogurt samples, including taste, odor, color, texture, and overall acceptability. The panelists used the 5‐point Hedonic scale test, where 1 represented a very poor sample and 5 a very good sample. Following coding, yogurt samples were given to panelists in plastic cups (Rahmani et al. 2021).

### 2.11 Statistical Analysis

All tests were repeated three times, and the data were expressed as means ± std. Statistical analysis of data was performed using one‐way analysis of variance (ANOVA) using SPSS 22.0 followed by Duncan multiply post hoc test at *p* < 0.05.

## 3 Results and Discussion

### 3.1 Characteristics and AA of CLN

Particle size is one of the key parameters in the application of nanoparticles in the food industry, because particle size affects the stability of colloidal systems, the bioavailability of compounds and their release rate as well as the organoleptic properties of food products enriched with them (Ballesteros et al. 2017). The mean particle size of curcumin nanocapsules prepared with Persian gum in this research was 237.2 nm, and these nanocapsules generally had a small size. The PDI of the curcumin nanocapsules was 0.219. In general, particles with PDI < 0.3 have favorable particle size dispersion (Table 1). Because it shows the degree of charge accumulation in the immobile layer and the strength of opposite ion adsorption on the particle surface, zeta potential is the best indicator for assessing the surface electrical condition of particles. For this reason, zeta potential is frequently used when reporting particle charge. Colloidal particles have a high zeta potential, which raises the electrostatic repulsion force and, in turn, the system's physical stability (Khoshmanzar et al. 2013). Often the zeta potential values higher than 30 mV (positive or negative) is considered as an indicator for optimal stability and reduction of aggregation and particle cohesion (Guerra‐Rosas et al. 2016). The curcumin nanocapsules prepared in the present study also had a negative surface charge and a high zeta potential (−30.78 mV), so they are physically stable. These nanocapsules also had high EE (96.84%) and were able to retain curcumin to a high extent in their structure. A hydrogen bond is established between the hydrocolloid's carboxyl groups and curcumin's hydroxyl groups, and also the lipophilic areas of the gum with the aromatic rings of the curcumin structure can establish a hydrophobic reaction, so the curcumin is well‐trapped in the coating and a high encapsulation efficiency is achieved.

**TABLE 1:** Characteristics of curcumin nano‐encapsulated with Persian gum.

Particle size (nm)	PDI	Zeta potential (mV)	EE (%)	DPPH (%)	FRAP (mM TE/g)
237.2 ± 8.9	0.219 ± 0.007	−30.78 ± 3.02	96.84 ± 2.76	80.79 ± 2.48	0.048 ± 0.001

*Note:* Values represent mean (*n* = 3) ± SD.

Abbreviations: EE: Encapsulation efficiency; FRAP: Ferric reducing ability of plasma; PDI: Polydispersity index.

**FIGURE 1:**
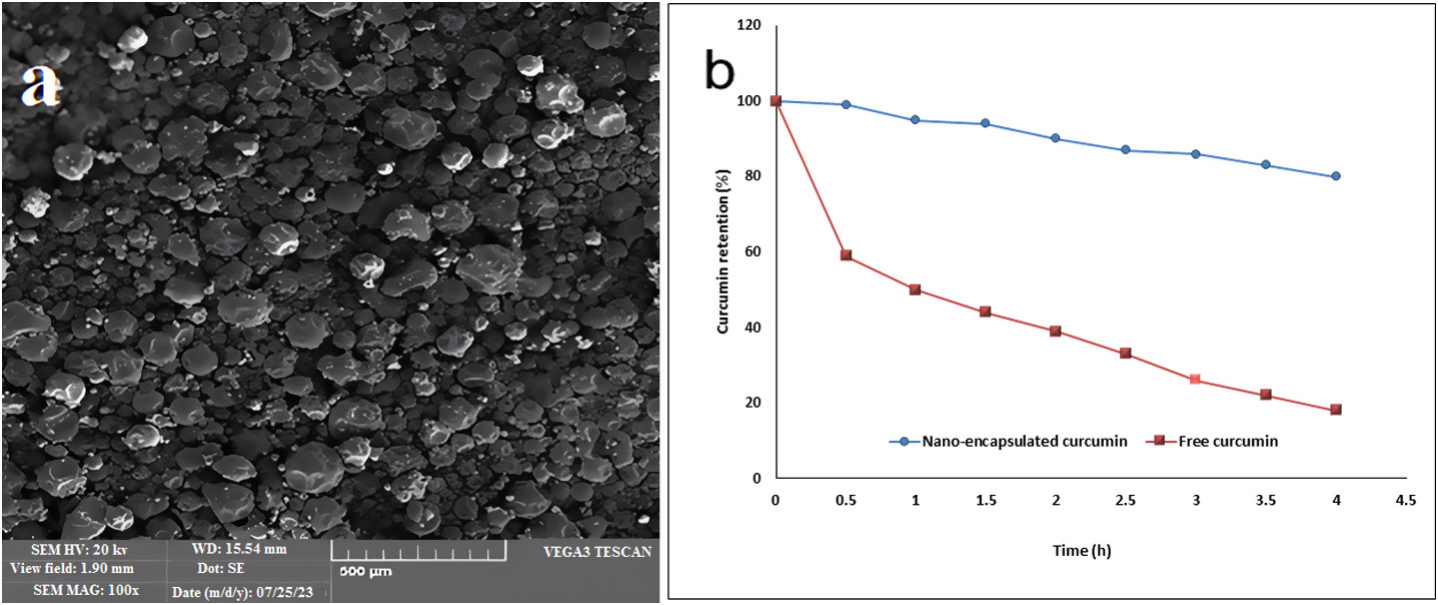
(a) SEM image of CLN prepared with Persian gum, and (b) the percentage of CLN prepared with Persian gum in release medium during 4 h of storage.

The CLN prepared in this research showed good AA, and their DPPH radical scavenging and FRAP were 80.79% and 0.048 mM TE/g, respectively. Ershadi et al. (2021) found that the nano‐encapsulation process could improve the AA of curcumin, and in their research the radical scavenging activity and reducing power values of CLN with Grass pea protein isolate/*Alyssum homolocarpum* gum were 73.27% and 0.22, respectively. The SEM image of CLN prepared with PG is given in Figure 1a. These nanocapsules generally had an irregular spherical shape and non‐uniform surfaces, and no accumulation of particles was observed in them. The main reason for the non‐accumulation is the negative charge of particles and the high value of zeta potential of CLN produced in this research that by creating a repulsive force, particles have been prevented from joining together. PG acts as a barrier that acts as a physical barrier with the coating itself, limiting the diffusion of foreign agents (e.g., microorganisms, enzymes) to the coated material (Dehghani et al. 2018).

### 3.2 Curcumin Retention Ability

The ability to retain curcumin by CLN is shown in Figure 1b. According to the figure, at the beginning of the experiment, the amount of curcumin in all samples was 100% and gradually during 4 h of storage in phosphate buffer with neutral pH, the amount of curcumin gradually decreased. As expected, the highest intensity of reduction of curcumin was related to curcumin without capsules (free curcumin), which showed the highest rate of reduction in the first 0.5 h of storage. Microencapsulation increased the stability of curcumin and significantly reduced the intensity of the decrease in the amount of this bioactive compound during the storage period. Despite the high functionalization activities of curcumin, these bioactive compounds are sensitive to light, oxygen, ionic strength, irradiation, and heat. When a coherent structure is placed around the active compound, it reduces the penetration of destructive factors to the center and core of the particles, thereby exerting its protective effect. Therefore, the nanoparticles produced in this research were able to increase the stability of curcumin against different conditions and showed high stability. The high stability of colloidal emulsions prepared with PG has been reported by different researchers. Meng et al. (2021) agreed that the nanoencapsulation of curcumin in the combination of zein and carboxymethyldextrin as a wall material could effectively increase the stability compared to free curcumin. Ershadi et al. (2021) in the study of the effect of nanoencapsulation with PG on the stability of curcumin observed that the gum coating method was able to increase the curcumin's stability against radiation, low pH, and hydrogen peroxide, while free curcumin exhibited the lowest stability. In comparison to the study of Dehghani et al. (2018) which investigated the antioxidant activity of clove and thyme essential oils coated in PG using the DPPH assay, the high DPPH% of clove and thyme essential oils reported is primarily attributed to the role of PG theological properties to maintain the phenolic compounds. PG or Farsi gum is a complex polysaccharide with a heterogeneous structure, containing both water‐soluble and water‐insoluble fractions. The water‐insoluble fraction of PG is primarily composed of high molecular weight polysaccharides with strong intermolecular interactions, resulting in a rigid and insoluble structure.

### 3.3 pH and Acidity

**FIGURE 2:**
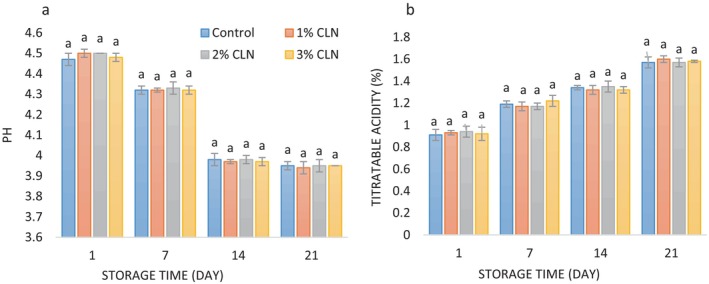
(a) Changes in pH values, and (b) changes in titratable acidity values (%) of stirred yogurt samples containing different levels of CLN during the cold storage period.

The average pH and acidity values of yogurt samples with varying concentrations of curcumin nanoparticles throughout the course of the 21‐day chilled storage are shown in Figure 2a,b. The average pH and acidity of the yogurt samples did not vary significantly over the course of the investigation. Due to lactose fermentation, acid generation, and starting bacterial activity, there was a fall in pH and an increase in acidity (*p* < 0.05) during storage. On the first day of the experiment, the pH and acidity values were the lowest (4.47–4.5 and 0.91%–0.94%, respectively), and on the last day of storage, the highest values (3.94–3.95 and 1.57%–1.60%, respectively). Similar findings of decreased pH and increased acidity in yogurt samples during storage have been reported by other researchers (Moghadam, Ariaii, and Ahmady 2021; Mohamed Ahmed et al. 2021; Zhao, Feng, and Mao 2020). PG affects pH by indirectly reducing microbial activity, preventing the formation of alkaline compounds (ammonia, amines) that would otherwise increase pH. The lack of effect of curcumin on the pH and acidity of dairy products such as cheese and yogurt has also been demonstrated by El‐Sayed and Shalaby (2021) and Shalaby and Amin (2018).

### 3.4 Starter Bacteria Viability

**FIGURE 3:**
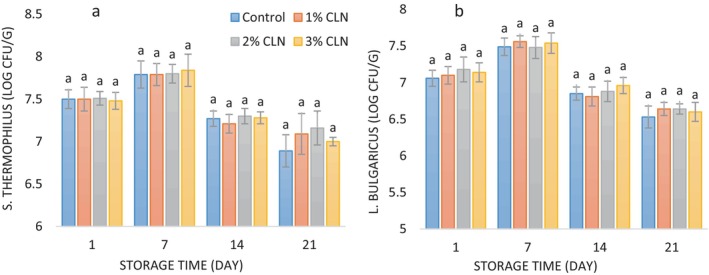
(a) Changes in *L. bulgaricus* counts (log CFU/g), and (b) changes in *S. thermophiles* counts (log CFU/g) of stirred yogurt samples containing different levels of CLN during the cold storage period.

The two main strains of bacteria used to start yogurt are *Lactobacillus delbrueckii* subsp. *Bulgaricus* and *Streptococcus thermophilus*. Lactic acid and formic acid are produced when carbohydrates are fermented by *Streptococcus* bacteria. The *Lactobacillus* bacteria are encouraged to proliferate by these acids. Yogurt's flavor and scent are derived from chemicals produced by the development of Lactobacillus, including acetaldehyde and diacetyl (Ahmad et al. 2021). The average count of *S. thermophilus* and *L. bulgaricus* varies during refrigerated storage in both the control yogurt and samples with varying amounts of CLN, as shown in Figure 3a,b. In every yogurt sample, the number of S. *thermophilus* stayed greater than the number of *L. bulgaricus* during the storage period. The mean number of yogurt starter bacteria in all of the samples—including those with different nanoparticle concentrations—did not change significantly. The addition of nanoparticles did not significantly affect the viability of the starter bacteria. These findings align with previous research by Rahmani et al. (2021), Maleki, Ariaii, and Sharifi Soltani (2021), and Niamah, Al‐Sahlany, and Al‐Manhel (2016). The count of starter bacteria rose initially throughout the course of the 21‐day storage period before declining until the last day of refrigeration (*p* < 0.05). Shalaby and Amin 2018 also observed a one‐log decrease in the number of starter bacteria during a 14‐day cold storage period; however, the addition of red cabbage and turmeric extracts had no discernible impact on the quantity of starter bacteria in the yogurt samples. Jozve‐Zargarabadi, Fadaei‐Noghani, and Huseini (2020) and Šeregelj et al. (2019) similarly reported no impact of phenolic compounds on the growth and viability of starter and lactic acid bacteria in yogurt during refrigerated storage. Typically, the decline in the count of starter bacteria starting from the seventh day onward is attributed to increased acid production and pH reduction, creating an unfavorable environment for their growth and survival. Furthermore, the reduction in nutrient availability in the samples and the production of other by‐products by the starter bacteria could contribute to the decrease in their count during storage (Jozve‐Zargarabadi, Fadaei‐Noghani, and Huseini 2020; Wang et al. 2022).

In general, the health benefits of yogurt are attributed to its nutrient composition and the presence of starter and probiotic bacteria. To maximize these health benefits, it is recommended to ensure that the count of starter and probiotic bacteria falls within the recommended range of 6–8 log CFU/g (Ross et al. 2005). According to the results of this study, despite the decrease in the number of starter bacteria during cold storage, however, until the last day of storage, the number of these bacteria was in acceptable range for health benefits. The numbers of *S. thermophilus* and *L. bulgaricus* in different yogurt samples were in the range of 6.89–7.84 log CFU/g and 6.53–7.56 log CFU/g, respectively. PG demonstrated varying protective effects on different microorganisms. While in the study of Mohammadi‐Gouraji, Sheikh‐Zeinoddin, and Soleimanian‐Zad (2017) it showed no significant impact on *L. plantarum* survival at 1%, a 6% concentration provided comparable protection to gum arabic for *E. coli*. This suggests that PG could be a suitable alternative to gum arabic for preserving certain bacteria, but its effectiveness might depend on the specific microorganism and concentration used.

### 3.5 Syneresis and WHC

**FIGURE 4:**
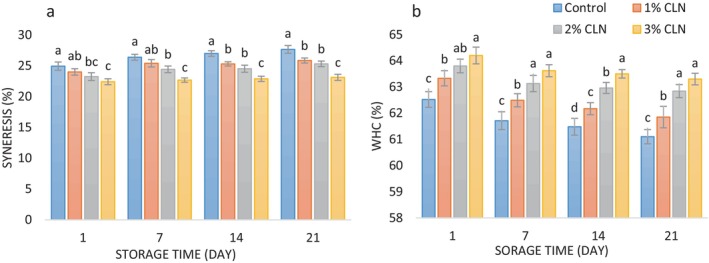
(a) Changes in syneresis values (%), and (b) changes in WHC (%) values of stirred yogurt samples containing different levels of CLN during the cold storage period.

Syneresis, the separation of the aqueous phase from the gel network, is a major drawback in yogurt production. While it is desirable in cheese making, it is unfavorable in yogurt production. Syneresis in yogurt occurs due to the contraction of the protein network structure, which weakens the binding capacity of whey proteins, resulting in their release from the yogurt (Sahan, Yasar, and Hayaloglu 2008; Varedesara, Ariaii, and Hesari 2021). Figure 4a illustrates the syneresis percentage of control yogurt and samples containing various levels of curcumin nanoparticles during 21‐day cold storage. The addition of curcumin nanoparticles at levels ranging from 1% to 3% in the yogurt formulation resulted in a significant decrease in syneresis percentage (*p* < 0.05). On the first day of the experiment, the control sample exhibited the highest syneresis value (42.93%), while the sample containing 3% curcumin nanoparticles showed the lowest value (22.40%). Gholamhosseinpour and Zare (2024) reported these reasons for the role of PG in syneresis reduction. PG as a water‐binding agent: By increasing the water‐holding capacity of the fermented dairy products (Wu et al. 2024), PG reduces the amount of free water available for separation, thereby minimizing syneresis. Modification of protein structure: PG interacts with milk proteins, forming complexes that create a more stable and cohesive protein network, preventing the release of whey and reducing syneresis. Increased viscosity: The higher viscosity imparted by PG to the product matrix hinders the movement of whey particles, preventing their aggregation and subsequent expulsion as syneresis.

The reduced syneresis in yogurt due to the addition of nanocapsules is likely attributed to the increased dry matter content and improved water holding capacity of the yogurt, which reduces the amount of separated serum. Akgün et al. (2020) also observed decreased syneresis in yogurt with the incorporation of chitosan‐coated cherry extract liposomes. Similarly, Yekta and Ansari (2019) found that the inclusion of hydrocolloids, such as gums, in the yogurt formulation can reduce syneresis by enhancing water holding capacity and viscosity.

The yogurt samples' syneresis levels rose with time, although the control sample's increase was more pronounced than that of the other samples. The control sample had a syneresis value of 27.64% on the final day of cold storage, whereas samples containing 1%, 2%, and 3% of curcumin nanocapsules had values of 25.84%, 25.30%, and 23.11%, respectively. The progressive loosening of the yogurt texture and the release of water attached to the proteins are responsible for the increase in syneresis during storage. By encouraging protein denaturation, pH lowering significantly contributes to the syneresis of yogurt samples during storage (Staffolo et al. 2004). Furthermore, rearrangement of the milk casein network during storage contributes to increased syneresis in yogurt (Ramirez‐Santiago et al. 2010). Despite the gradual increase in syneresis during cold storage, the naked eye may not easily perceive these changes, and they do not significantly affect the appearance of the yogurt samples. Similar findings of gradual syneresis increase in yogurt samples during cold storage have been reported by Maleki, Ariaii, and Sharifi Soltani (2021), Rahmani et al. (2021), and Zhao, Feng, and Mao (2020).

Water holding capacity (WHC) is an essential parameter that impacts the quality of yogurt. It refers to the ability of proteins in yogurt curd to retain water (Dinkçi et al. 2021). The results of WHC values for yogurt samples containing various levels of curcumin nanocapsules are depicted in Figure 4b. On the initial days of the experiment, the control sample exhibited the lowest WHC value (62.52%), and as the levels of nanocapsules increased in the samples, the WHC values also increased significantly (*p* < 0.05). The sample containing the highest level of nanocapsules demonstrated the highest WHC value on this day (64.20%). Overall, the addition of curcumin nanocapsules prepared with PG to the yogurt formulation increased the total solids content, consequently leading to an increase in WHC. These findings are consistent with previous studies (Niamah, Al‐Sahlany, and Al‐Manhel 2016; Yekta and Ansari 2019). During the 21‐day cold storage period, the WHC values of the yogurt samples decreased. However, the reduction in WHC was statistically significant only between the first and seventh days of storage (*p* < 0.05). On the last day of storage, the control sample exhibited the lowest WHC value (61.10%), while the sample containing 3% of curcumin nanocapsules showed the highest value (63.30%). Still, there was no significant distinction between this sample and the 2% nanocapsule‐containing yogurt (62.84%). In a study by El‐Said, El‐Messery, and El‐Din (2018), similar declines in WHC of yogurt samples appeared after a 20‐day cold storage period. Furthermore, El‐Messery et al. (2019) reported a decreased WHC of yogurt during a 15‐day cold storage period.

### 3.6 Apparent Viscosity

**FIGURE 5:**
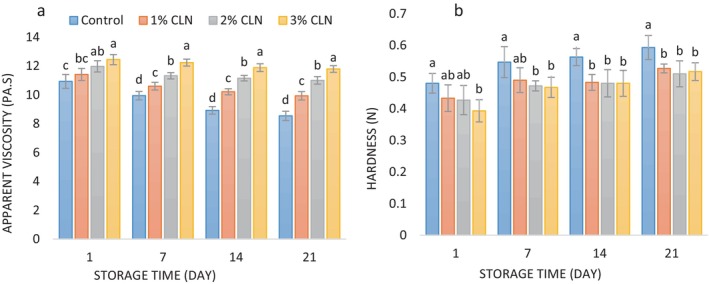
(a) Changes in apparent viscosity values (Pa.s), and (b) changes in hardness values (N) of stirred yogurt samples containing different levels of CLN during cold storage period.

Important yogurt quality parameters include the composition of raw milk, dry matter content, type of starter bacteria, heating duration and temperature, and storage conditions all affect viscosity. The apparent viscosity of samples with varying amounts of CNL and control stirred yogurt varies during the course of a 21‐day cold storage period, as shown in Figure 5a. Yogurt sample viscosity was shown to significantly decrease after storage (*p* < 0.05). Changes in the connections between proteins in the yogurt protein network are responsible for this decline (Boeneke and Aryana 2008), which eventually causes the samples' syneresis % to rise. The acidification of yogurt during storage, which results in a watery consistency and decreased viscosity, is another reason for the decrease in viscosity (Senadeera et al. 2018).

The yogurt samples' initial viscosities varied from 10.93 to 12.44 Pa, and on the last day of storage, they reached 8.54–11.79 Pa. Overall, there was a significant increase in perceived viscosity (*p* < 0.05) when CLN were added to yogurt samples, with amounts raised from 1% to 3%. The sample with the greatest concentration of nanocapsules (3%) showed the highest viscosity, whereas the control sample continuously showed the lowest viscosity throughout the investigation. Previous investigations have documented a reduction in the apparent viscosity of yogurt samples while stored at low temperatures (Jozve‐Zargarabadi, Fadaei‐Noghani, and Huseini 2020; Maleki, Ariaii, and Sharifi Soltani 2021; Moghadam, Ariaii, and Ahmady 2021; Varedesara, Ariaii, and Hesari 2021). In accordance with the results of the present study, Moghadam, Ariaii, and Ahmady (2021) also observed a remarkable improvement in the apparent viscosity of yogurt samples with the addition of encapsulated pennyroyal extract. Cho et al. (2020) also found that incorporating olive leaf extract to the formulation of yogurt reduced the syneresis percentage and increased the apparent viscosity of yogurt samples. Increased viscosity of yogurt samples in the presence of hydrocolloids such as gums was also reported by Yekta and Ansari (2019) and Amiri Aghdai, Alami, and Rezai (2010).

### 3.7 Hardness of Texture

Hardness is a significant textural parameter of yogurt, representing the minimum force required to compress the product. Figure 5b illustrates the hardness values of yogurt samples containing different levels of CLN. It can be observed that on the initial day of the experiment, the addition of nanocapsules to yogurt samples, facilitated by the presence of PG, resulted in decreased hardness values. The reason for this decline is that hydrocolloids include polysaccharides that can integrate into casein micelle structure and obstruct the development of a three‐dimensional protein network. On the other hand, PG reduces hardness by increasing water content and enhancing proteolysis. More moisture softens the product matrix while protein degradation further weakens the structure. While factors such as salt concentration, fat content, and pH increase hardness, PG counteracts these effects by retaining water and potentially affecting protein structure. Yogurt's tiny structure enlarges and widens as a result, decreasing its hardness (Yadmellat, Jooyandeh, and Hojati 2017).

On the other hand, the samples' hardness progressively rose with time (*p* < 0.05). On the first day of storage, the samples' hardness values varied from 0.393 N to 0.480 N, and on the last day, they reached 0.510 N to 0.593 N. The elimination of moisture from the samples and a decrease in water holding capacity are probably to blame for the rise in hardness throughout the storage period. Similar increases in hardness during cold storage were also observed by El‐Messery et al. (2019) and de Campo et al. (2019). El‐Sayed and Shalaby (2021) reported that the incorporation of curcumin nano‐emulsions into cheese formulations reduced hardness compared to the control, but hardness increased during storage. Likewise, Liu, Shi, and Li (2018) found that the addition of curcumin slightly reduced the hardness of yogurt samples.

### 3.8 Color Parameters

Color is a significant visual attribute of dairy products. Table 2 presents the variations in color parameters of stirred yogurt samples containing different levels of curcumin nanocapsules during a 21‐day cold storage period. On the initial day of the experiment, the control sample exhibited the highest L* value (88.49), indicating lightness, while the lowest values of a* (−1.94) and b* (9.35) denote redness and yellowness, respectively. As the levels of nanocapsules in the yogurt samples increased, the lightness decreased, and the yellowness and redness of the color intensified (*p* < 0.05), resulting in a more yellow coloration of the yogurt samples. Throughout the 21‐day storage period, the L* values decreased, while the a* and b* parameters gradually increased (*p* < 0.05).

**TABLE 2:** Changes in color parameters of stirred yogurt samples containing different levels of CLN during cold storage period.

Samples	Day1	Day 7	Day 14	Day 21
L* index				
Control	88.49 ± 0.76^A,a^	87.25 ± 0.53^AB,a^	86.45 ± 0.39^BC,a^	86.10 ± 0.47^C,a^
1% CLN	86.48 ± 0.37^A,b^	85.87 ± 0.44^AB,b^	85.23 ± 0.56^B,b^	85.00 ± 0.53^B,b^
2% CLN	86.10 ± 0.68^A,b^	85.48 ± 0.40^AB,b^	84.84 ± 0.49^B,b^	84.57 ± 0.51^B,b^
3% CLN	84.70 ± 0.62^A,c^	84.15 ± 0.48^AB,c^	83.84 ± 0.42^AB,c^	83.49 ± 0.45^B,c^
a* index				
Control	−1.94 ± 0.05^D,d^	−1.15 ± 0.13^C,d^	0.35 ± 0.21^B,d^	0.95 ± 0.10^A,d^
1% CLN	2.54 ± 0.07^D,c^	2.96 ± 0.10^C,c^	3.16 ± 0.04^B,c^	3.32 ± 0.08^A,c^
2% CLN	6.17 ± 0.09^C,b^	6.35 ± 0.07^B,b^	6.54 ± 0.09^A,b^	6.69 ± 0.11^A,b^
3% CLN	8.63 ± 0.12^C,a^	8.94 ± 0.09^B,a^	9.19 ± 0.10^AB,a^	9.30 ± 0.16^A,a^
b* index				
Control	9.35 ± 0.26^D,d^	10.08 ± 0.33^C,d^	10.85 ± 0.37^B,d^	11.59 ± 0.32^A,d^
1% CLN	17.19 ± 0.30^C,c^	17.84 ± 0.25^B,c^	18.38 ± 0.28^AB,c^	18.81 ± 0.21^A,c^
2% CLN	20.21 ± 0.23^C,b^	20.72 ± 0.19^B,b^	21.18 ± 0.34^AB,b^	21.67 ± 0.25^A,b^
3% CLN	24.14 ± 0.39^B,a^	24.84 ± 0.31^B,a^	25.62 ± 0.22^A,a^	26.15 ± 0.34^A,a^

*Note:* Values represent mean (*n* = 3) ± SD.

Abbreviation: CLN, curcumin‐loaded nanocapsules.

Marcolino et al. (2011) concurred that the addition of free curcumin and beta‐cyclodextrin‐curcumin complex to yogurt and cheese formulations led to a reduction in sample lightness. Almeida et al. (2018) found that incorporating free curcumin powder into yogurt decreased the L* value and increased the a* and b* values, whereas the use of curcumin nanocapsules prepared with PVP polymer did not alter the lightness of yogurt samples but increased the b* value and decreased the a* value. Sardiñas‐Valdés et al. (2021) also reported a decrease in lightness and an increase in yellowness in cheese color following the addition of nano‐emulsified curcumin.

### 3.9 AA of Yogurt

**FIGURE 6:**
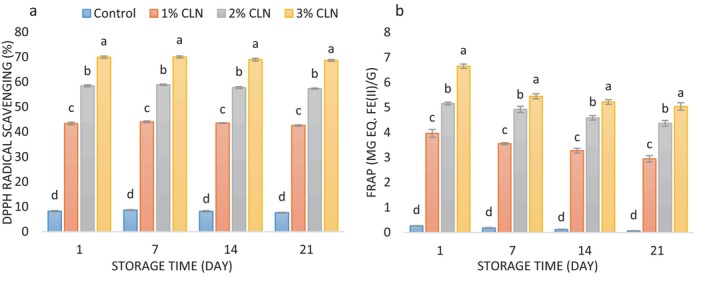
(a) Changes in DPPH radical scavenging values (%), and (b) changes in FRAP values (mg eq. Fe (II)/g) of stirred yogurt samples containing different levels of CLN during the cold storage period.

Using the DPPH radical scavenging and FRAP techniques, the AA of samples with varying concentrations of curcumin nanocapsules was evaluated; the findings are shown in Figure 6a,b, respectively. As a polyphenol component, curcumin is well‐known for its substantial AA (Pan et al. 2019). As a result, the yogurt formulation's AA of the fortified samples increased significantly (*p* < 0.05) in comparison to the control sample after adding CLN at increasing levels (1% to 3%). The control sample's DPPH radical scavenging and FRAP values, for example, were 8.20% and 0.264 mg Fe(II)/g on the first day of the experiment, respectively, while the sample with the highest concentration of nanocapsules (3%) had values that increased to 69.95% and 6.649 mg Fe(II)/g, respectively. Previous researchers have reported similar results. Liu, Shi, and Li (2018) demonstrated that the addition of curcumin increased the AA of yogurt samples as assessed by the FRAP and ABTS methods. Abdelrazik and Elshaghabee (2021) also observed increased AA in fermented cow milk and soy milk due to the addition of curcumin capsules. Sardiñas‐Valdés et al. (2021) reported an increase in the AA of cheese following the addition of curcumin.

Yogurt samples' ability to scavenge DPPH radicals rose from the first to the seventh day of the cold storage period; however, this shift was not statistically significant. However, the AA progressively dropped (*p* < 0.05) from the seventh to the last day of storage. Likewise, during the course of the 21‐day cold storage period, the samples' FRAP values steadily dropped (*p* < 0.05). The deterioration of phenolic chemicals over time, especially by lactic acid bacteria, is probably the cause of the drop in AA (Kim et al. 2019). Additionally, milk proteins can react with phenolic compounds and form complexes, thereby reducing the overall AA (Oksuz et al. 2019). The decrease in AA during the storage period has also been reported by other researchers (Ahmad et al. 2021; Durmus, Capanoglu, and Kilic‐Akyilmaz 2021; Maleki, Ariaii, and Sharifi Soltani 2021; Moghadam, Ariaii, and Ahmady 2021). The DPPH radical scavenging and FRAP values of yogurt samples on the final day of cold storage varied from 7.60% to 68.65% and from 0.058 mg Fe(II)/g to 5.040 mg Fe(II)/g, respectively.

### 3.10 Sensory Evaluation

**FIGURE 7:**
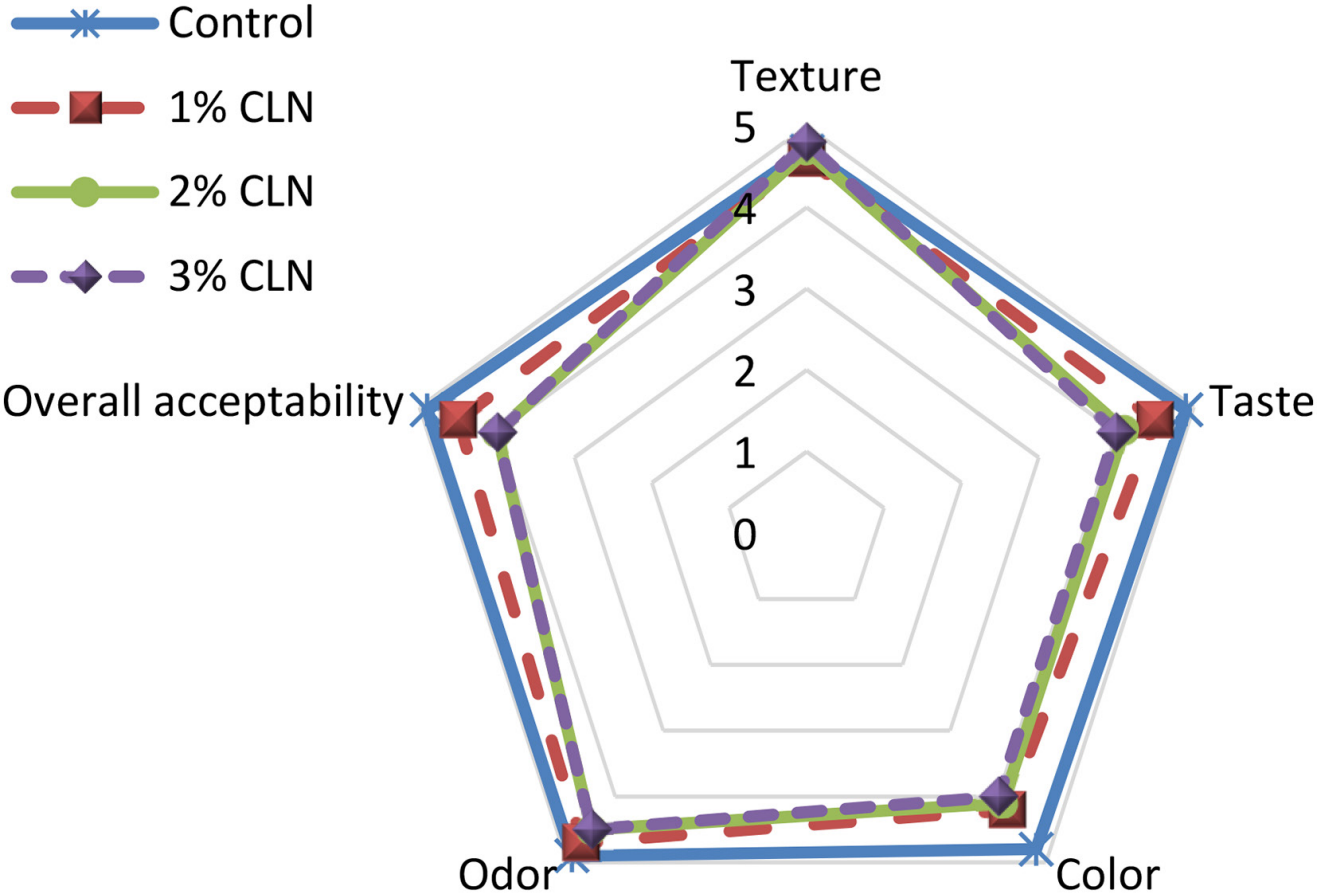
Comparison of sensory properties of stirred yogurt samples containing different levels of CLN.

A 5‐point Hedonic scale test was used to assess the texture, taste, color, odor, and overall acceptability of yogurt samples with varying concentrations of CLN. The findings are shown in Figure 7. The samples' texture and odor ratings did not significantly alter when CLN were added to the yogurt mixture. But the acceptance scores for color, taste, and overall reduction were all lower (*p* < 0.05). In terms of sensory features, all samples were deemed satisfactory and received good scores, even if these traits had decreased. PG was used to encapsulate curcumin, which resulted in a decrease in the compound's flavor, color, and odor intensity. The current study's results are consistent with those of El‐Sayed and Shalaby (2021), who observed that cheese samples containing 2.5% curcumin nano‐emulsion had similar sensory acceptance compared to the control sample, while samples containing 5% nano‐emulsion had lower sensory scores that were still generally acceptable. Liu, Shi, and Li (2018) also reported a reduction in the taste score of yogurt with the incorporation of curcumin (at a level of 0.5%), but there were no significant changes in the odor and texture scores of the samples, and the fortified sample was still considered generally acceptable. Kumar et al. (2016) found that the addition of curcumin nanocapsules prepared with sodium alginate did not have an adverse effect on the sensory properties of ice cream.

## 4 Conclusion

This study looked at the quality and antioxidant properties of Persian gum‐prepared curcumin nanocapsules added to stirred yogurt. The outcomes demonstrated that the pH, acidity, and viability of the yogurt starter bacteria were unaffected by the addition of curcumin nanocapsules. But in contrast to the control sample, it resulted in higher water holding capacity (WHC), apparent viscosity, hardness, and AA, and lower yogurt syneresis %. The yogurt samples that included CLN had a little yellower hue in comparison to the yogurt control. All of the yogurt samples had strong sensory ratings and were deemed acceptable, despite the inclusion of nanocapsules causing a drop in flavor, color, and overall acceptability of the samples. The use of Persian gum‐prepared curcumin nanocapsules enables the creation of functional fermented dairy products with possible health advantages, according to the study's findings.

## Author Contributions

**Maziar Taghadosi:** formal analysis (equal), investigation (equal), methodology (equal), writing – original draft (equal). **Marzieh Bolandi:** conceptualization (equal), data curation (equal), supervision (equal), writing – review and editing (equal). **Homa Baghaei:** data curation (equal), supervision (equal), writing – review and editing (equal).

## Ethics Statement

The study was reviewed and approved by the Islamic Azad University of Damghan branch and informed consent was obtained from each subject prior to their participation in the study.

## Consent

The authors have nothing to report.

## Conflicts of Interest

We thus declare that none of the authors has published the study's findings before and that they are not being considered for publication anywhere. The writers state that they are not aware of any conflicts of interest.

## Data Availability

The data are available upon reasonable request.
